# Phrenology

**Published:** 1848-07

**Authors:** 


### PHRENOLOGY.

Phrenologists say that when the bump of acquisitiveness is too largely developed it produces a strong propensity to steal whenever a temptation is offered. Now, if any of our brethren should be thus unfortunately organized, and in filling a tooth should be tempted to save their gold by filling the bottom of the cavity with tin or any cheap metal, we would advise them, if possible, to change the current of the propensity, and direct the hand to the pocket, hat or handkerchief or any thing else. It is true there is rather more danger of present detection. But just explain the nature of the case to them and I have no doubt they will let you pass, by far preferring to lose a few dollars on their hat or even their cloak, than their tooth by malpractice. Honesty is the best policy even among thieves. E. T.

